# Oportunidades perdidas: contatos de adolescentes vítimas de homicídio com instituições do Estado

**DOI:** 10.1590/0102-311XPT120224

**Published:** 2025-04-25

**Authors:** Marcelo Ryngelblum, Maria Fernanda Tourinho Peres

**Affiliations:** 1 Faculdade de Medicina, Universidade de São Paulo, São Paulo, Brasil.

**Keywords:** Adolescente, Violência com Arma de Fogo, Violência, Homicídio, Colaboração Intersetorial, Adolescent, Gun Violence, Violence, Homicide, Intersectoral Collaboration, Adolescente, Violencia con Armas, Violencia, Homicidio, Colaboración Intersectorial

## Abstract

No Brasil, os homicídios têm afetado profundamente os padrões de mortalidade desde os anos 1980, com impactos significativos na população adolescente. Este estudo tem como objetivo descrever os contatos dos adolescentes (10 a 19 anos) vítimas de homicídio (2015 a 2020) e as instituições públicas no município de São Paulo, destacando as vulnerabilidades programáticas e oportunidades perdidas de prevenção. Utilizando a metodologia de record *linkage*, descrevemos os contatos institucionais dos adolescentes nas políticas públicas (saúde, educação, assistência social, justiça e segurança pública). Nossos resultados indicam que as vítimas de homicídio eram predominantemente do sexo masculino (94%) e negras (67%), com baixos níveis de escolaridade e alta taxa de abandono escolar (68%), vivendo em situação de vulnerabilidade socioeconômica. A maioria tinha histórico de conflito com a lei (57%) e, entre esses, 69,2% cumpriram medida de internação socioeducativa na Fundação Casa. A principal causa de morte entre os adolescentes no período foi a atividade policial (55%). Ao mapear os contatos institucionais dos adolescentes nossa pesquisa identifica oportunidades perdidas de prevenção. A colaboração intersetorial poderia reduzir significativamente os homicídios de adolescentes, enfatizando a necessidade de abordagens abrangentes, baseadas em direitos, para enfrentar as vulnerabilidades sociais. Apesar dos avanços sociais notáveis, São Paulo, a cidade mais rica do Brasil, continua a apresentar marcada desigualdade, evidente nas diferentes perspectivas de vida de seus habitantes.

## Abstract (en)

In Brazil, homicides have profoundly affected mortality patterns since the 1980s, with significant impacts on the adolescent population. This study aims to describe the contact between adolescent (10 and 19 years old) victims of homicide (2015 to 2020) and public institutions in the Municipality of São Paulo, highlighting programmatic vulnerabilities and missed opportunities for prevention. Using the record linkage methodology, we describe the institutional contact of adolescents with public policies (health, education, social services, justice and public security). Our results indicate that homicide victims were predominantly male (94%) and black (67%), with low levels of education and high school dropout rate (68%), living in a situation of socioeconomic vulnerability. Most had a history of conflict with the law (57%) and, among these, 69.2% served a socio-educational detention measure at Casa Foundation. The main cause of death among adolescents in the period was police activity (55%). By mapping adolescents’ institutional contact, our research identifies missed opportunities for prevention. Intersectoral collaboration could significantly reduce adolescent homicides by emphasizing the need for comprehensive, rights-based approaches to address social vulnerabilities. Despite remarkable social advances, São Paulo, the richest city in Brazil, continues to present marked inequality, evident in the different perspectives of life of its inhabitants.

## Abstract (es)

En Brasil, los homicidios han afectado profundamente los patrones de mortalidad desde los años 1980, con impactos significativos en la población adolescente. Este estudio tiene como objetivo describir los contactos de adolescentes (10 y 19 años) víctimas de homicidio (2015 a 2020) y las instituciones públicas en el Municipio de São Paulo, con foco en las vulnerabilidades programáticas y las oportunidades perdidas para la prevención. A partir de la metodología de vinculación de registros, describimos los contactos institucionales de los adolescentes en las políticas públicas (salud, educación, asistencia social, justicia y seguridad pública). Nuestros resultados indican que las víctimas de homicidio fueron predominantemente del sexo masculino (94%) y negras (67%), con bajos niveles de estudios y una alta tasa de deserción escolar (68%), y que viven en una situación de vulnerabilidad socioeconómica. La mayoría tenía antecedentes de conflicto con la ley (57%); y, entre estos, el 69,2% cumplió con una medida de reclusión socioeducativa en la Fundación Casa. La principal causa de muerte entre los adolescentes en el período fue la actividad policial (55%). Al mapear los contactos institucionales de los adolescentes, nuestro estudio identifica oportunidades perdidas para la prevención. La colaboración intersectorial podría reducir significativamente los homicidios de adolescentes, lo que destaca la necesidad de enfoques integrales, basados en los derechos para hacer frente a las vulnerabilidades sociales. A pesar de los notables avances sociales, São Paulo, la ciudad más rica de Brasil, sigue mostrando una marcada desigualdad, evidente en las diferentes perspectivas de vida de sus habitantes.

## Introdução

Desde 1996, a Organização Mundial da Saúde (OMS) reconhece a violência como um dos temas prioritários de saúde global, e recomenda a criação de medidas para a prevenção e mitigação dos seus efeitos ^1^. Embora a violência não seja objeto restrito e específico da área da saúde, é crescente a participação do setor na formulação de questões, reflexões e respostas ao problema ^2^.

O Brasil desde os anos de 1980 vem vivenciando uma mudança significativa nos padrões de mortalidade. As causas externas, que incluem as mortes por violência, passaram a ocupar a segunda posição entre as causas de óbito no país. A transição epidemiológica se deu com aumento expressivo dos casos de homicídio - “o grande inimigo da saúde pública” ^3^, tornando-se uma problemática prioritária para o campo da saúde. Desde os anos 1980 vê-se o aumento dos homicídios no Brasil, que foram de 14 mil óbitos em 1980 para cerca de 60 mil em 2020, em sua maioria cometidos por arma de fogo ^4^, posicionando o Brasil como um dos países com as maiores taxas de homicídio no mundo ^5^.

Segundo os dados do Ministério da Saúde ^6^, a violência interpessoal continua como uma das principais causas de óbito no país, ocupando a sexta posição entre todas as causas, e a primeira entre adolescentes e jovens de 15 a 29 anos, em especial os do sexo masculino. Nesse grupo, em 2018, a taxa de homicídios atingiu 112,4 óbitos/100 mil habitantes ^4^. No Município de São Paulo nos últimos dez anos, segundo o Comitê Paulista de Prevenção ao Homicídio na Adolescência (CPPHA), 2.359 adolescentes foram a óbito em decorrência dos homicídios.

No período de 2009 a 2019, adolescentes (10 a 19 anos), 7% da população residente no Município de São Paulo, representaram 17,5% das mortes violentas intencionais ocorridas na cidade. Em comparação com as taxas de homicídio de outros grupos etários, nota-se que os adolescentes entre 2015 e 2018 foram o segundo grupo com a maior taxa de mortalidade por homicídio no Município de São Paulo, estando atrás somente dos jovens com idade de 20 a 29 anos, revelando a especial gravidade da violência direcionada a esse público.

Fenômeno complexo, a violência na adolescência é resultado da interação entre múltiplos fatores de risco que incluem características dos sujeitos, suas relações e o contexto em que estão inseridos ^7^. Uma série de fatores de risco são reconhecidos como preditores da violência na adolescência, entre os quais destacamos ^7^ ^,^ ^8^: o baixo rendimento e o abandono escolar; experiências de abuso ou negligência na infância; exposição a violência comunitária ou no ambiente doméstico; o envolvimento com crime e histórico de conflito com a lei; a pobreza e desigualdade socioeconômica; a precariedade de residência; e o desemprego, a dificuldade de geração de renda e a inserção precária em contextos de trabalho informais e ilegais. Cabe afirmar, que nenhum fator isolado explica a vitimização ou a perpetração de atos violentos, tampouco a presença de fatores de risco significa que o adolescente vivenciará, necessariamente, a violência em sua vida.

As mortes de adolescentes por violência interpessoais intencionais ocorrem em um contexto de profundas iniquidades. Nesse sentido, o homicídio, nesse grupo, pode ser compreendido como o resultado final de uma série de processos de vulnerabilizações e exclusões enfrentados por esses adolescentes em seu processo de desenvolvimento. Especial atenção deve ser dada à vulnerabilidade programática ^9^, que representa as falhas das políticas estatais em promover proteção integral às crianças e adolescentes, seja garantindo o acesso e a permanência educacional, seja propiciando amplo acesso a serviços de saúde, promovendo a inclusão social e prevenindo o cometimento de atos infracionais, por exemplo. Como afirmam Galdeano & Almeida ^10^, o Estado, por meio da vulnerabilidade programática, está presente em todas as etapas do desenvolvimento de crianças e adolescentes, seja pela perpetuação da segregação e precariedade urbana, seja pela política educacional deficitária, pela atenção à saúde insuficiente, ou atuação policial seletiva que produz mortes e encarceramentos.

Cabe, no entanto, investigar as múltiplas nuances da presença do Estado na vida desses adolescentes e suas famílias, ampliando o entendimento da atuação institucional que ocorre de maneira muitas vezes contraditória e que se dá em um emaranhado complexo de políticas sociais e de repressão. Conforme Rui ^11^ analisa, não cabe questionar se há ausência do Estado, mas sim de destrinchar a atuação Estatal em que coexistem processos de punição e cuidado, repressão e políticas sociais. Nesse sentido, o nosso interesse recai na descrição da circulação dos adolescentes vítimas de homicídios, em sua maioria marginalizados e provenientes das periferias, nos equipamentos públicos de cuidado, assistência, educação e punição, com vistas a descrever, analisar e apreender os múltiplos pontos de contato com a burocracia Estatal que, se analisados em conjunto, podem contribuir para a prevenção dessas mortes. Na medida em que o nosso foco são adolescentes vítimas de homicídios, consideramos esses pontos de contato como oportunidades perdidas pelo Estado, que por meio da atuação intersetorial, poderia coibir a violação de direitos das crianças e adolescentes, e prevenir a sua morte violenta.

Procuraremos mapear os contatos dos adolescentes vítimas de homicídio com as diversas instituições públicas responsáveis pelo Sistema de Garantia de Direitos da Criança e do Adolescente (SGDCA). O SGDCA representa a convergência estratégica entre governos e sociedade civil com o propósito de efetivar os direitos das crianças e dos adolescentes ^12^. Assim, os registros oficiais das instituições públicas podem explicitar situações de vulnerabilidade social, e podem ser considerados registros de oportunidades perdidas de atuação e prevenção, constituindo-se como marcadores que podem ser utilizados, de forma sistemática, para o delineamento de planos de prevenção a mortes violentas, se identificados oportunamente.

A nossa hipótese é que adolescentes que estabelecem contato com instituições que compõem o SGDCA apresentam maiores marcas de vulnerabilização. Cabe ressaltar que não concebemos esse contato como um fator de risco ou como etiologicamente associado ao homicídio, mas sim como um indicador de situações de vida em que a garantia de direitos está comprometida.

Assim, buscamos descrever o contato de adolescentes (de 10 a 19 anos) residentes no Município de São Paulo, vítimas de homicídio (2015 a 2020) com instituições que compõem o SGDCA e sinalizam para situação de exclusão e vulnerabilidade programática.

## Metodologia

O delineamento deste trabalho pode ser descrito como um estudo epidemiológico descritivo dos contatos institucionais dos adolescentes que morreram por homicídio no Município de São Paulo, entre os anos de 2015 e 2020, realizado a partir de dados secundários.

Todos os adolescentes de 10 até 19 anos que foram a óbito por homicídios nos anos de 2015 a 2020 no Município de São Paulo foram identificados. Neste estudo, consideramos como morte por homicídio todas as mortes classificadas como homicídio doloso, latrocínio, lesão corporal seguida de morte e morte decorrente de intervenção policial, a partir dos registros da Secretaria de Segurança Pública do Estado de São Paulo (SSP-SP).

### Fontes das informações

#### Secretaria de Segurança Pública do Estado de São Paulo

Solicitamos à SSP-SP uma base de dados com informações sobre todos os casos de homicídio doloso, latrocínio, lesão corporal seguida de morte e morte decorrente de intervenção policial, registrados no Município de São Paulo entre 2015 e 2020 e cuja vítima tivesse entre 10 e 19 anos. As seguintes informações foram solicitadas e disponibilizadas: data registro, categoria, número do boletim de ocorrência, circunscrição, departamento circunscrição, cidade, delegacia, data fato, hora fato, tipo de local, logradouro, latitude e longitude, tipo pessoa, sexo, idade, data de nascimento, número de documentos, raça/cor, profissão, nome completo, nome mãe.

#### Sistema de Informações sobre Mortalidade

Foi solicitado ao Programa de Aprimoramento das Informações de Mortalidade (PROAIM) da Secretaria Municipal da Saúde de São Paulo, o gestor municipal do Sistema de Informações sobre Mortalidade (SIM), uma base de dados contendo todos os óbitos de adolescentes residentes e ocorridos no Município de São Paulo nos anos de 2015 a 2020, classificados em sua causa básica como agressões (X85-Y09), eventos cuja intencionalidade é indeterminada (Y10-Y34), intervenções legais e operações de guerra (Y35-Y36), acidentes (V01-X59), causas mal definidas e não especificadas (R99) e lesões autoprovocadas intencionalmente (X60-X84). A classificação da causa básica de óbito segue a 10ª revisão da Classificação Internacional de Doenças (CID-10) ^13^. As seguintes informações foram solicitadas e disponibilizadas: numeração da Declaração de Óbito (DO), data do óbito, nome completo da vítima, nome da mãe da vítima, data de nascimento, idade da vítima, logradouro de moradia da vítima, município de moradia da vítima, bairro/distrito de moradia da vítima, Registro Geral (RG), endereço da ocorrência, bairro/distrito da ocorrência, local de ocorrência do óbito, profissão (ocupação habitual e ramo de atividade), escolaridade, sexo, raça/cor.

#### Informações de agravos de notificação 

No Brasil, a violência é um evento de notificação pelos profissionais de saúde de todos os níveis de atenção desde 2007. Isso significa que, em tese, todos os casos de violência devem ser notificados ao Sistema de Informação para a Vigilância de Violências e Acidentes (SIVVA), de 2007 até 2015 e ao Sistema de Informação de Agravos de Notificação (SINAN), a partir de 2015 em diante, com especial importância para crianças e adolescentes. Foram solicitadas à Secretaria Municipal da Saúde de São Paulo as seguintes informações da ficha de notificação: dados gerais, notificação individual, dados de residência, dados da pessoa atendida, dados da ocorrência, violência, violência sexual, dados do autor da violência, evolução, encaminhamento e data do registro/notificação.

#### Cadastro Único da Assistência Social

O Cadastro Único da Assistência Social (CadÚnico) identifica todas as famílias com renda mensal de até meio salário mínimo por pessoa. Famílias com renda superior poderão ser incluídas no CadÚnico, desde que sua inclusão esteja vinculada à seleção ou ao acompanhamento de programas sociais. Solicitamos as seguintes informações à Secretaria Municipal de Assistência Social de São Paulo: identificação e características das pessoas, características do domicílio, informações sobre geração de renda.

#### Secretaria Estadual de Educação de São Paulo

Solicitamos para a Secretaria Estadual de Educação de São Paulo (SEDUC) o banco de dados de matrícula escolar por aluno desde a primeira matrícula no Ensino Fundamental I até a última matrícula escolar registrada, englobando dados de 2002 a 2020. Foram solicitadas as seguintes informações: nome do aluno, nome dos pais, código aluno, código escola, data de nascimento, rendimento escolar, deficiência, raça/cor, bairro de residência, código de endereçamento postal (CEP) do aluno, tipo de ensino, série, Bolsa Família, data matrícula, data início matrícula, data final matrícula, situação do aluno (ativo, transferido, reclassificado etc.), falecimento do aluno, data de falecimento, informação sobre frequência escolar.

#### Tribunal de Justiça de São Paulo

Solicitamos ao Tribunal de Justiça de São Paulo (TJ-SP) o histórico infracional das vítimas objetivando identificar aqueles adolescentes que tiveram contato com a justiça, com a ocorrência ou não de medida socioeducativa. Buscamos com o TJ-SP apenas a existência ou não de histórico de conflito com a lei (sim ou não).

#### Fundação Casa

Foi requisitado o histórico infracional e institucional dos casos que cumpriram medida socioeducativa de internação. As informações solicitadas foram: número do prontuário da Fundação Casa, existência de outras medidas socioeducativas de internação pelo adolescente (sim ou não), quantidade de passagens pela Fundação Casa, datas de entrada e saída da primeira ou última internação.

### Vinculação dos bancos de dados

Duas estratégias foram utilizadas para a vinculação dos dados. A primeira estratégia foi a utilização da vinculação probabilística (*record linkage*). A segunda estratégia, que se impôs no decorrer da pesquisa, foi o envio de listas contendo o nome e outras informações pessoais dos adolescentes incluídos para que as secretarias realizassem as buscas em seus bancos de dados. Essa estratégia foi necessária diante da recusa em fornecer a base completa, sendo a disponibilização dos dados autorizada a partir de uma listagem prévia. No Quadro 1 sistematizamos as estratégias utilizadas para a vinculação dos bancos de dados.

**Quadro 1:** Sistematização das estratégias de vinculação de dados.

BANCOS DE DADOS	METODOLOGIA	RESPONSÁVEL PELA VINCULAÇÃO
SSP-SP e TJ-SP + Fundação Casa (conflito com a lei)	Lista	TJ-SP e Fundação Casa
SSP-SP e SIM/PROAIM	*Record linkage*	PROAIM
SSP-SP e SINAN/SIVVA	*Record linkage*	Autores
SSP-SP e SEDUC	*Record linkage*	Autores
SSP-SP e CadÚnico	*Record linkage*	Autores

CadÚnico: Cadastro Único da Assistência Social; PROAIM: Programa de Aprimoramento das Informações de Mortalidade; SEDUC: Secretaria Estadual de Educação de São Paulo; SINAN: Sistema de Informação sobre Agravos de Notificação; SIVVA: Sistema de Informação para a Vigilância de Violências e Acidentes; SSP-SP: Secretaria de Segurança Pública do Estado de São Paulo; TJ-SP: Tribunal de Justiça de São Paulo.

A vinculação entre as bases da SSP-SP e do SIM foi realizada por servidores do Programa de Aprimoramento das Informações de Mortalidade, Coordenação de Epidemiologia e Informação (PROAIM-CEINFO) da Secretaria Municipal de Saúde de São Paulo, a partir da metodologia de *record linkage*. A busca das vítimas nos dados do TJ-SP e da Fundação Casa foi realizado pelos servidores das instituições a partir de uma listagem enviada, contendo: nome completo da vítima, data de nascimento e nome completo da mãe. Após o envio, os servidores devolveram a planilha aos pesquisadores contendo as informações solicitadas que foram adicionadas ao banco de dados.

Os pesquisadores realizaram o *record linkage* nos bancos de dados do SINAN/SIVVA, educação e CadÚnico utilizando o pacote *RecordLinkage* ^14^ desenvolvido para o software RStudio (https://rstudio.com/). Nos bancos de dados do SINAN/SIVVA e da educação utilizou-se a chave de blocagem o *soundex* do primeiro nome da vítima e seu último sobrenome, a data de nascimento e o *soundex* do primeiro nome da mãe.

No banco de dados do CadÚnico, iniciamos nossa busca nos cadastros ativos do Estado de São Paulo entre 2015 e 2020 e nos cadastros inativos por motivo de óbitos do Município de São Paulo para os mesmos anos. Estratégias de pareamento determinísticas e probabilísticas via *record linkage* foram utilizadas. Inicialmente utilizamos estratégias de vinculações determinísticas utilizando Cadastro de Pessoa Física (CPF), Número de Inscrição Social (NIS) ou RG dos adolescentes. Posteriormente, técnicas de vinculação probabilísticas (*record linkage*) foram aplicadas, utilizando em um primeiro momento os dados das vítimas e posteriormente os dados das mães das vítimas. Para vinculação com o CadÚnico utilizamos uma combinação de informações das vítimas e, nos casos em que esses não foram encontrados, de familiares de primeiro grau.

Por fim, após as vinculações trabalhou-se com um banco de dados unificado contendo todas as informações das vítimas.

### Análise

Foi realizada análise descritiva dos dados com cálculo de medidas de tendência central e dispersão para as variáveis quantitativas, e proporções para as variáveis categóricas. Todas as análises foram realizadas no software RStudio.

### Aspectos éticos

O estudo foi aprovado pelo Comitê de Ética em Pesquisa da Faculdade de Medicina, Universidade de São Paulo (FMUSP, protocolo nº 5.647.223) e pela Secretaria Municipal da Saúde de São Paulo (protocolo n º 5.683.265). Todas as secretarias envolvidas no estudo concederam autorização para o acesso aos dados e manifestaram concordância com a condução da pesquisa. Além disso, a pesquisa recebeu a aprovação do Poder Judiciário do Estado de São Paulo, garantindo a conformidade ética e legal necessária para sua realização.

## Resultados

No período entre 2015 e 2020 foram registrados pela SSP-SP 1.036 casos de homicídios de adolescentes com idade entre 10 e 19 anos, residentes e ocorridos no Município de São Paulo. A principal causa de homicídios, segundo a SSP-SP, é a morte decorrente de intervenção policial, representando mais de 50% dos casos, seguido dos homicídios dolosos (43,44%), os latrocínios (1,35%) e a lesão corporal seguida de morte (0,29%). Considerando a causa básica de óbito constante na DO, vemos que 57,43% (n = 595) dos casos foram classificados como morte por agressão (X85-Y09), seguida dos eventos cuja intenção é indeterminada (Y10-Y34) com 20,66% (n = 214) e das intervenções legais e operações de guerra (Y35-Y36) com 17,76% (n = 184). Representando menos do que 5% estão as outras categorias. Cabe destacar que segundo os dados do SIM, 85,91% (n = 890) dos óbitos foram cometidos com armas de fogo.

Na Tabela 1 vemos as características sociodemográficas dos adolescentes. Embora haja alguma diferença, as características sociodemográficas das vítimas são muito semelhantes nas duas fontes. Segundo as fontes, as vítimas possuíam em média 17 anos de idade. Cerca de 50% veio a óbito com até 17/18 anos de idade e 25% possuía até 16 anos de idade quando faleceu. Ao analisarmos o campo raça/cor, percebe-se que as vítimas em sua maioria são negras (negros e pardos), representando 66,86% no SIM e 68,53% na SSP-SP. As vítimas brancas variaram entre 29% na SSP-SP e 32,95% no SIM. Cerca de 94% das vítimas eram do sexo masculino.

**Tabela 1:** Característica sociodemográfica de adolescentes vítimas de homicídio no Município de São Paulo, Brasil (Sistema de Informação sobre Mortalidade - SIM e Secretaria de Segurança Pública do Estado de São Paulo - SSP-SP), 2015-2020.

Características	SSP-SP	SIM (saúde)
n (%)	n (%)
Idade (anos)		
Média (DP)	17,33 (1,46)	17,36 (1,48)
Mediana (IIQ)	17 (16-19)	18 (16-19)
Raça/Cor		
Branca	302 (29,15)	340 (32,95)
Indígena	-	2 (0,19)
Parda	607 (58,59)	593 (57,46)
Negra	103 (9,94)	97 (9,40)
Outras	16 (1,54)	-
Sem informação *	8	4
Sexo		
Feminino	61 (6,03)	59 (5,69)
Masculino	972 (93,97)	977 (94,31)
Sem informação *	3	-
Estado civil		
Solteiro	-	1.018 (99,03)
Casado	-	3 (0,29)
Viúvo	-	1 (0,10)
Divorciado	-	-
União estável	-	5 (0,49)
Sem informação *	-	9

DP: desvio padrão; IIQ: intervalo interquartil.

* Optou-se por não utilizar os valores percentuais na categoria “sem informação”.

Quando consideramos os locais de moradia dos adolescentes vítimas de homicídio, após a realização do georreferenciamento, cerca de 60% viviam em locais com altas taxas de homicídio (> 10,52 óbitos/100 mil). Somente 3,45% das vítimas viviam em locais com taxas muito baixas (< 5,8 óbitos/100 mil) - dados não apresentados em tabela.

Foram encontrados 77 (7,43%) adolescentes com ao menos uma notificação de violência. Em oito adolescentes duas notificações foram realizadas, totalizando 85 notificações de violência contra as vítimas no período. Chama a atenção que 40 notificações (47,06%) ocorreram ou no mesmo dia do evento fatal, ou em datas muito próximas (um a três dias). Nas 45 (52,94%) restantes, o tempo da notificação até a morte foi de dois anos e meio, sendo que em 25% dos casos o óbito ocorreu em até cinco meses. A média de idade das notificações de violência foi de 16 anos, com a menor idade encontrada de sete anos. Os homens são a maioria dos casos notificados (97,1%). As violências mais notificadas foram a física (n = 76; 89,41%) sendo que os meios de agressão mais comumente empregados foram as armas de fogo (n = 46; 54,12%), a força corporal (n = 26; 30,59%) e os objetos perfurocortantes (n = 8; 9,41%). Em 13 casos (15,3%) as agressões foram cometidas por agentes institucionais (policiais e funcionários da Fundação Casa), em 11 (12,95%) o agressor era familiar e em dois casos foram os companheiros e ex-companheiros (2,36%). Em oito casos (9,41%) constata-se violência de repetição e em 19 casos (22,35%) houve mais do que um agressor. Cabe mencionar a baixa qualidade dos dados do sistema de notificação de violências, com muitos dados faltantes e preenchimento não padronizado dos campos.

A maioria dos adolescentes vítimas de homicídios possuía histórico de conflito com a lei (57,05%; n = 591). Desses, 123 (20,81%) obtiveram remissão ou arquivamento do processo. Chama a atenção a desproporção racial das vítimas que possuíam histórico de conflito com a lei, entre as quais 70,06% eram negras. A polícia foi a principal responsável pelas mortes daqueles com histórico de conflito com a lei (64,13% de morte decorrente de intervenção policial).

Na Tabela 2 apresentamos os dados de medida socioeducativa com restrição de liberdade: 409 (69,2%) vítimas cumpriram medida socioeducativa em regime de internação ou semiliberdade, indicando o uso frequente desse tipo de medida pelos operadores do Direito. Em média, a idade de início da medida de internação foi 16,3 anos e a de término, 16,8 anos. O número de internações variou entre uma e sete com 50% dos adolescentes cumprindo múltiplas medidas de restrição de liberdade. A duração média de internação foi de 175 dias. É possível notar que a duração média aumenta conforme a reincidência, de 103 dias para 343 dias. O tempo máximo decretado foi de 1.055 dias.

**Tabela 2:** Medidas de internação na Fundação Casa entre adolescentes vítimas de homicídios no Município de São Paulo, Brasil (2015-2020).

Características das medidas de restrição de liberdade (Fundação Casa)	Média (DP)	Mediana (IIQ)	Mínimo-Máximo
Idade de entrada (anos) *	16,3 (1)	16,5 (15,6-17,2)	12,9-19
Idade de saída (anos) *	16,8 (1,2)	16,9 (16-17,8)	12,9-19,2
Tempo de internação (dias) *	175 (193)	65 (28-299)	0-1.055
Número de internações (dias) *	1,84 (1,1)	1 (1-2)	1-7
1	103 (152)	36 (18-146)	0-806
2-3	230 (196)	224 (44-360)	0-1.055
4-7	343 (210)	353 (166-458)	36-1.002
Número de internações	n (%)		
1	198 (50,13)		
2	112 (28,35)		
3	51 (12,91)		
4	24 (6,08)		
5	5 (1,27)		
6	3 (0,76)		
7	2 (0,51)		
Tempo decorrido até o óbito após a última medida socioeducativa com restrição de liberdade (dias)	Média (DP)	Mediana (IIQ)	Mínimo-Máximo
Total	418 (358)	323 (149-594)	0-2.331
1 internação	481,8 (414,1)	372 (160-689)	0-2.331
2-3 internações *	368,3 (293,5)	306 (129-532)	0-1.415
4-7 internações *	298,0 (225,1)	228 (127-384)	34-858

DP: desvio padrão; IIQ: intervalo interquartil.

* Em caso de múltiplas internações foi considerada a mais recente.

Na Tabela 2, nota-se que os homicídios acontecem em 418 dias após a desinternação, sendo que pelo menos a metade ocorre em até um ano após a saída da instituição. Os adolescentes com maior quantidade de passagens pela Fundação Casa morreram mais rapidamente após a última medida, com destaque para adolescentes que possuíam de quatro a sete internações em que cerca de 75% morrem em pouco mais de um ano após a desinternação (384 dias).

Dos 1.036 adolescentes, conseguimos identificar 939 (90,64%) na base de dados da SEDUC. Noventa e sete casos (9,36%) foram excluídos por não terem sido localizados. No momento do óbito, a maioria (67,84%; n = 637) já havia abandonado a escola. Cerca de 24,71% (n = 232) estavam matriculados, e 70 (7,45%) tinham completado o processo escolar, com Ensino Médio completo. A maioria possuía Ensino Médio incompleto (n = 407; 43,34%), seguido de Ensino Fundamental incompleto (n = 388; 41,32%), Ensino Fundamental completo (n = 74; 7,88%) e Ensino Médio completo (n = 70; 7,45%).

Chama a atenção que 64,53% dos adolescentes tinha alguma reprovação em sua trajetória escolar, com uma média de 1,64 reprovações. Daqueles que tiveram reprovações, 45,21% tiveram duas ou mais reprovações. O principal motivo de reprovação dos estudantes foi a baixa frequência escolar (61,6%), seguida do baixo desempenho escolar (38,39%). Destaca-se que os principais ciclos escolares com reprovação foram o Ensino Fundamental nos anos finais (6º-9º anos) e o Ensino Médio com 40,49% e 35,2%, respectivamente.

A Tabela 3 apresenta os sucessivos processos de rupturas escolares e seus momentos. Como exposto anteriormente, a reprovação é um desfecho escolar bastante presente nas trajetórias escolares. Nota-se que em média do momento da primeira reprovação até a evasão existe um hiato breve de cerca de um ano, momento chave para intervenções. O tempo decorrido da primeira reprovação até o momento da morte é, em média, 2,8 anos, com 75% das mortes ocorrendo em até quatro anos após a primeira reprovação. Já o tempo entre a evasão e a morte é, em média, de um ano e meio, com 30% das vítimas falecendo em até seis meses e 75% das vítimas falecendo em até dois anos e meio após a evasão escolar, com idade média de 16,3 anos.

**Tabela 3:** Tempos de evasão, reprovação e morte de adolescente vítimas de homicídio. São Paulo, Brasil, 2018-2020.

Variáveis	Média (DP)	Mediana (IIQ)	Mínimo-Máximo	n
Tempo primeira retenção-evasão (anos)	1,1 (1,85)	0 (0-2)	0-9	493
Tempo primeira retenção-morte (anos)	2,8 (2,45)	2,3 (1-4)	0-13	606
Tempo evasão/última matrícula-morte (anos)	1,8 (1,7)	1,3 (0,5-2,6)	0-9	637
Idade da evasão (anos)	16,3 (1,9)	16,5 (15,2-17,7)	10-19	637

DP: desvio padrão; IIQ: intervalo interquartil.

Nota: abandono/última matrícula-morte (anos): até 6 meses [192 (30,14%)]; 6 meses e 1 dia-12 meses/1 ano [89 (13,97%)]; 1 ano-2 anos [141 (22,14%)]; 3 anos-5 anos [80 (12,56%)]; > 5 anos [36 (5,65%)].

Os dados do CadÚnico dão um panorama das características socioeconômicas das vítimas e suas famílias. Quinhentas e sessenta e duas vítimas (54,35%) estavam registradas no CadÚnico e 268 (25,87%) recebiam o Bolsa Família. Observa-se que em média a renda das famílias das vítimas constantes no CadÚnico foi de R$ 583,00 com uma mediana de R$ 300,00 (intervalo interquartil - IIQ: 50-984; mínimo: R$ 0,00, máximo: R$ 7.280,00). Cerca de 20% (n = 69) das famílias declararam não possuir nenhuma renda. Em média, as casas possuem um dormitório e três cômodos, e em cerca de 75% das casas vivem mais do que quatro pessoas por domicílio, com uma média de 3,5 pessoas por domicílio. Em 98% das residências a composição familiar se restringe a apenas uma família habitando o mesmo domicílio. Segundo os dados, 1,36% (n = 7) das vítimas eram familiares de pessoas encarceradas e 1,91% (n = 10) vinham de famílias de catadores de materiais recicláveis. Dos grupos populacionais tradicionais, apenas uma vítima era indígena.

## Discussão

Nesse estudo, o nosso objetivo foi, a partir da vinculação de bases de dados intersetoriais, descrever os contatos de adolescentes vítimas de homicídios com diversas instituições que compõem o SGDCA no Brasil. O SGDCA tem por missão proteger crianças e adolescentes e garantir acesso a direitos que culminem em sua segurança e desenvolvimento saudável. Esses objetivos devem ser alcançados por meio de políticas públicas e da atuação de instituições como escolas, unidades de saúde, sistema de justiça e programas sociais. Dessa forma, nos casos de adolescentes assassinados, esses contatos prévios com instituições do sistema de garantia de direitos foram considerados como oportunidades perdidas da garantia da segurança e prevenção dos homicídios. A vinculação das bases de dados mostrou-se uma estratégia viável para aprofundar o conhecimento e identificar marcadores de vulnerabilidade dos adolescentes aos homicídios, podendo contribuir para formulação de políticas de prevenção.

Vale destacar que a maioria dos homicídios aconteceram em decorrência da atividade policial, sendo esta a principal causa de mortalidade para os adolescentes no Município de São Paulo após o cruzamento dos dados. No Brasil, discute-se que a atuação violenta é um componente estruturante das dos policiais decorrente da sua militarização. O perfil racial das vítimas, com desproporcional presença da população negra, especialmente aqueles com histórico de atos infracionais gera preocupação. A violências praticada contra a população negra explicita o racismo estrutural brasileiro, que é o organizador das desigualdades no país ^15^. Nossos resultados apoiam fortemente a afirmação do racismo, como um aspecto central da estrutura social brasileira, que impulsiona a brutalidade policial e influencia o funcionamento do sistema de justiça ^16^ ^,^ ^17^ ^,^ ^18^.

Segundo Pequeno ^19^, a confluência de classe social, raça e território articulam desigualdades e a distribuição desproporcional da violência letal, recaindo sobre os adolescentes pobres, negros e periféricos os processos de estigmatização, segregação e que culminam, por fim, nos homicídios. Nesse sentido, certas representações sociais da adolescência enquanto “problema”, “perigo”, vinculadas a transgressão, apresentam consequências práticas que resultam na criminalização de certos tipos de adolescências no Brasil. Essas interpretações estão para além de uma mera representação social inócua, mas se materializam socialmente e institucionalmente na forma pelo qual o Estado lida com questão da adolescência. Como Rizzini ^20^ discute, a ambivalência com que a atenção à infância e juventude se constituiu historicamente no Brasil se expressa na dicotomização entre a infância em risco e a infância perigosa. Essa dicotomia, que gerou acesso à cidadania para os primeiros e a tutela, a vigilância e o controle do Estado para os últimos, se perpetua como fio histórico até os dias de hoje.

Nota-se que a absoluta maioria dos homicídios de adolescentes no Município de São Paulo foram cometidas por armas de fogo (85,91%). Esse resultado deve ser um sinal de alerta para as recentes iniciativas para flexibilização do porte e posse de arma no Brasil, uma vez que as evidências vão na direção de apontar uma relação direta entre circulação de armas e aumento dos homicídios ^21^, com impactos específicos nessa população.

Chamou nossa atenção o pequeno número de notificações de violência pelos profissionais de saúde, considerando que estas são compulsórias no caso de crianças e adolescentes. Além disso, o preenchimento da ficha de notificação apresenta problemas, como informações faltantes, que aponta para a necessidade de esforços para a melhoria do sistema de notificação. Sabe-se que os sistemas de informação que monitoram as violências são estratégicos para a sua prevenção ^22^. Ainda que qualquer conclusão a respeito dos padrões de vitimização e sua relação com o setor saúde fiquem comprometidos, alguns pontos merecem ser explorados. Destacamos, a precocidade de violência nas trajetórias de vida destes adolescentes e as dinâmicas de violências repetitivas.

Quase metade das notificações encontradas no sistema de informação se originaram nas unidades de saúde que prestaram atendimento à violência que resultou no óbito. É possível supor que esses foram casos notificados de violência grave, possivelmente cometidos com armas de fogo. No entanto, os demais casos, cuja notificação não se relaciona à violência letal, a média de tempo entre a notificação e a morte foi de dois anos e cerca de um quarto aconteceu em até cinco meses após a notificação. Esses representam mais da metade dos casos notificados, sendo oportunidades perdidas de prevenção.

Mais da metade dos adolescentes assassinados e analisados neste artigo tiveram histórico de conflito com a lei ou algum contato com o sistema de justiça. Uma parcela significativa deles cumpriu medida socioeducativa com restrição de liberdade na Fundação Casa. Ainda que o sistema socioeducativo seja um avanço nas práticas e discursos sobre a menoridade, o que se observa é a precariedade do sistema prisional para adolescentes infratores e a reprodução da violência nas situações de internação ^10^ ^,^ ^23^. Os resultados evidenciam a manutenção da violência na vida dos adolescentes, sendo os períodos pós-internação fundamentais para a prevenção dos homicídios. Aos nossos dados, somam-se a literatura que aponta a estreita vinculação entre experiências de conflito com a lei, o contato com o sistema de justiça e o risco aumentado de morte violenta ^8^ ^,^ ^24^ ^,^ ^25^ ^,^ ^26^.

Apenas um quarto dos adolescentes estava matriculado na escola no momento do óbito. A maior parte dos adolescentes já tinha abandonado a escola no momento do óbito (68%), percentual de evasão muito acima do observado para o Estado de São Paulo ^27^ (1,7% no anos finais do Ensino Fundamental e 5,3% no Ensino Médio em 2021). Percentuais semelhantes de evasão foram observados no Ceará com adolescentes vítimas de homicídio em que 70% desses estavam há pelo menos seis meses fora da escola ^8^. Ambos os percentuais denotam a face perversa da evasão escolar no Brasil: aprofunda as desigualdades, reproduz processos de exclusão e amplifica o risco de morte violenta precoce.

Estudos têm apontado que os principais motivos de evasão escolar no Brasil são a necessidade de gerar renda e o desinteresse pelo processo escolar ^8^ ^,^ ^28^. No Brasil, a entrada para o mundo do trabalho nessa fase da vida se dá mediante relações precarizadas, em posições de pior remuneração, com exposição a riscos ^10^, e em detrimento do processo escolar. Se a emergência da adolescência está identificada com a universalização escolar e com o afastamento dos adolescentes do mundo do trabalho ^29^, nos questionamos em que medida existe uma experiência comum de adolescência no contexto brasileiro, marcado pela desigualdade social, onde grupos sociais são excluídos do processo escolar e ingressam precocemente no mundo do trabalho.

A fragilização do processo de escolarização se faz perceber também nas trajetórias escolares irregulares dos adolescentes vítimas de homicídio. A maioria dos adolescentes tinha reprovação em sua trajetória escolar. Sabe-se que crianças e adolescentes em atraso escolar, para além das implicações futuras da perda do processo de aprendizagem, também estão mais vulneráveis à violência ^30^. O principal motivo das reprovações foi a frequência, que precisa ser compreendido dentro de uma dinâmica de ruptura com o processo escolar, que pode levar anos, e é antecedida por sinais que, quando oportunamente reconhecidos, possibilitam intervenções. Destacamos que o absenteísmo, que gera reprovação por frequência, e que pode culminar, por fim, na própria evasão escolar, expressa sucessivas rupturas institucionais.

Os dados do CadÚnico jogam luz na condição de pobreza e extrema pobreza das vítimas de homicídio e suas famílias, revelando um percentual expressivo de famílias vivendo em condições adversas. Ainda que a maioria das famílias tenha condições adequadas de moradia, merece destaque a alta presença de violência nos distritos de moradia das vítimas, com taxas muito altas de homicídios. É necessário refletir sobre o fato desses adolescentes e suas famílias se desenvolverem e socializarem em um ambiente marcado pela insegurança e pelo medo, com suas repercussões físicas, psíquicas e desenvolvimentais.

Os resultados evidenciam falhas nas políticas públicas, manifestadas como vulnerabilidades programáticas na vida dos adolescentes. Dialogando com Rui ^11^, não se trata de questionar ou sugerir uma suposta ausência estatal, uma vez que a presença do Estado em todas essas etapas é incontestável. No entanto, essa presença, assim como a mera expansão dos serviços públicos, revela-se insuficiente para assegurar, de forma efetiva, a garantia dos direitos dos adolescentes.

## Considerações finais

No Brasil, a geração na qual este estudo se debruça, aquela nascida entre 1996 e 2005, foi impactada por um conjunto de conquistas na efetivação da garantia de direitos, nascendo em condições de vida mais vantajosas se comparado a gerações anteriores. Embora os avanços sociais sejam inegáveis, o Município de São Paulo, o mais rico do país, mantém acentuada desigualdade que se expressa como um abismo entre perspectivas de vida.

Os resultados apontam para um perfil bastante específico das vítimas: em sua maioria homens negros, com baixa escolaridade, que evadiram do processo escolar, com trajetórias de conflito com a lei, vivendo situações de vulnerabilidade socioeconômica.

Nossa experiência com a ligação dos bancos de dados aponta para o potencial do compartilhamento de informações entre as secretarias com vistas a prevenir a morte violenta intencional na adolescência. A junção das informações que estão dispersas nas bases de dados da administração pública pode ser um caminho para o diálogo e elaboração de planos de ação comuns. O compartilhamento e a corresponsabilização pelos adolescentes é necessária em um cenário tão desafiador.

Busca-se, futuramente, expandir o escopo dos serviços analisados, incorporando a trajetória dos jovens na Rede de Atenção à Saúde, com destaque para a Rede de Atenção Psicossocial no acompanhamento de crianças e adolescentes em sofrimento psíquico, frequentemente relacionado ao uso problemático de álcool e outras drogas, e à violência. Inclui-se, ainda, os Centros de Referência Especializados de Assistência Social (CREAS) no atendimento a casos de violação de direitos e as medidas socioeducativas em meio aberto.

Por fim, algumas limitações devem ser consideradas, como a impossibilidade de encontrar todas as trajetórias escolares, em que cerca de 10% das vítimas foram excluídas.
